# Factors affecting public access defibrillator placement decisions in the United Kingdom: A survey study

**DOI:** 10.1016/j.resplu.2022.100348

**Published:** 2023-01-07

**Authors:** Diane Lac, Maria K. Wolters, K.H. Benjamin Leung, Lisa MacInnes, Gareth R. Clegg

**Affiliations:** a Resuscitation Research Group, The University of Edinburgh, Edinburgh, Scotland, United Kingdom; b School of Informatics, The University of Edinburgh, Edinburgh, Scotland, United Kingdom; c Department of Mechanical and Industrial Engineering, University of Toronto, Toronto, Ontario, Canada; d Scottish Ambulance Service, Edinburgh, Scotland, United Kingdom

**Keywords:** Public access defibrillator, Automated external defibrillator, Out-of-hospital cardiac arrest, Bystanders, Resuscitation, Defibrillation

## Abstract

**Aim:**

This study aimed to understand current community PAD placement strategies and identify factors which influence PAD placement decision-making in the United Kingdom (UK).

**Methods:**

Individuals, groups and organisations involved in PAD placement in the UK were invited to participate in an online survey collecting demographic information, facilitators and barriers to community PAD placement and information used to decide where a PAD is installed in their experiences. Survey responses were analysed through descriptive statistical analysis and thematic analysis.

**Results:**

There were 106 included responses. Distance from another PAD (66%) and availability of a power source (63%) were most frequently used when respondents are deciding where best to install a PAD and historical occurrence of cardiac arrest (29%) was used the least. Three main themes were identified influencing PAD placement: (i) the relationship between the community and PADs emphasising community engagement to create buy-in; (ii) practical barriers and facilitators to PAD placement including securing consent, powering the cabinet, accessibility, security, funding, and guardianship; and (iii) ‘risk assessment’ methods to estimate the need for PADs including areas of high footfall, population density and type, areas experiencing health inequalities, areas with delayed ambulance response and current PAD provision.

**Conclusion:**

Decision-makers want to install PADs in locations that maximise impact and benefit to the community, but this can be constrained by numerous social and infrastructural factors. The best location to install a PAD depends on local context; work is required to determine how to overcome barriers to optimal community PAD placement.

## Introduction

Each year in the United Kingdom (UK), the National Health Service treats over 30,000 out-of-hospital cardiac arrests (OHCAs) with about 8% of patients surviving to hospital discharge.^1^, ^2^, ^3^, ^4^, ^5^ Prompt bystander-initiated defibrillation using public access defibrillators (PADs) can be an effective intervention for OHCA patients.^6^, ^7^, ^8^, ^9^, ^10^, ^11^ Bystander defibrillation before the arrival of emergency medical services can increase survival by up to sevenfold.^8^, ^12^, ^13^, ^14^, ^15^, ^16^, ^17^

To maximise the likelihood of PAD usage they need to be rapidly accessible in the event of an OHCA. Recommendations from the American Heart Association have suggested that PADs should be available within 1.5 minutes’ “brisk walk” from an OHCA.^18^ However, studies show that often no PADs are available, or that they are located far from where OHCAs are likely to occur^19^, ^20^, ^21^, ^22^; PAD inaccessibility leads to lower usage by the public and is associated with poorer survival outcomes.^18^, ^20^, ^23^ This highlights the need for approaches to encourage placement of PADs in locations where they are most likely to be available to treat OHCA.

This study used a web-based survey to examine current community PAD placement strategies employed by both individuals and organisations in the UK, identify factors which influence PAD placement decision-making and investigate how each factor plays a role in determining the final location of a PAD.

## Methods

Ethical approval for this study was obtained through the School of Informatics at the University of Edinburgh (RT number #6166).

### Study design

A web-based survey complaint with the CHERRIES guidelines^24^ was conducted and consisted of multiple choice, Likert scale, and free-text questions designed to investigate current PAD placement strategies employed by individuals, groups, and organisations in the UK. This survey collected data as part of a larger research study to develop a digital support tool to guide PAD placement decision-making using a data-driven approach. This paper reports on survey questions collecting demographic information, facilitators and barriers to community PAD placement and information used to decide PAD placement. The survey was developed by the investigator team and piloted with the Stakeholder Advisory Board consisting of subject matter experts and representatives of national organisations involved in PAD placement for content validity and clarity of language. Further refinements were made to reach the final set of questions. The survey was hosted by Online Surveys (https://www.onlinesurveys.ac.uk/, Bristol, UK).

### Study recruitment

A purposive sample was collected via email invitations and social media advertising through the networks of the Save a Life for Scotland (SALFS) partnership (www.savealife.scot) and the Stakeholder Advisory Board. Participants were invited to take part in the survey if they were involved in the process of placing PADs within the UK and were able to respond in English. This includes individuals, community groups, charities, or organisations involved in placing PADs in the community, as well as those involved in policy development related to OHCA or PADs. Survey invitations were sent to the Stakeholder Advisory Board to capture their answers separately.

### Data analysis

Descriptive statistical analysis was completed using R (R Foundation for Statistical Computing). Qualitative data was analysed using Braun and Clarke’s six-phase framework^25^ to conduct a structured thematic analysis for all free-text answers via QSR Nvivo12 Plus (Burlington, MA, USA).

## Results

140 responses were received, including 10 responses from the Stakeholder Advisory Board. Thirty-four responses from social media advertising were excluded, consisting of 1 duplicate submission and 33 bot responses identified by free-text responses clearly unrelated to the survey area. This resulted in a total of 106 responses included in analysis. Characteristics of survey respondents are shown in Table 1. Individuals made up 32% of respondents with 68% replied on behalf of their organisation.

**Table 1:** Characteristics of survey respondents.

Which option best describes the organisation you represent when you are involved in placing PADs? Percentages are relative to the 72 respondents who self-identified as representing an organisation.	N	%
Commercial organisation	3	4
Emergency Services	7	10
Local community group	23	32
National third sector/charitable organisation	19	26
Public sector – central government	2	3
Public sector – local authority	5	7
Public sector - NHS	5	7
Other	8	11
Total	72	100
N/A – Not responding on behalf of an organisation	34	
**Where in the UK do you or your organisation place most of your PADs?**	**N**	**%**
England	19	18
Northern Ireland	4	4
Scotland	60	57
Wales	7	7
We don’t place PADs	10	9
We place PADs across the whole UK	6	6

Respondents were asked about the types of information they use when deciding where best to install a PAD. The results are shown in Table 2. Historical occurrence of OHCA was used the least frequently at 29% while distance from another PAD was used the most frequently at 66%.

**Table 2:** Types of information used by survey respondents always or often when deciding on PAD placement locations.

How often do you use the following types of information to decide the best place to install a PAD?	N	%
Distance from another PAD	70	66
Availability of a power source for a heated cabinet	67	63
Footfall in an area	65	61
Local knowledge of the person/organisation purchasing the PAD	65	61
Safety and security of the PAD	65	61
Population density in an area	64	60
Planning regulations	44	42
Historical occurrence of cardiac arrest	31	29

Examination of free-text responses about how PAD placement decisions are made revealed three main themes:

• The relationship between the community and PADs
• Practical barriers and facilitators to community PAD placement
• ‘Risk assessment’ methods used to estimate the need for PADs in a given location

### Relationship between the community and PADs

Respondents expressed the importance of engaging with communities and creating buy-in as a crucial element to successful and sustainable PAD placement. Two sub themes were identified:

### Community engagement and securing buy-in

Engagement with the community to create buy-in was identified as a key facilitator of PAD placement and the sustainability of a placed PAD. Methods used to engage with communities included:

• Speaking with the local community about the importance and benefits of PADs.
• Highlighting previous OHCA incidents in the area.
• Joint consultation to determine the best location to install the PAD.
• Identifying a PAD ‘guardian’ or ‘custodian’ within the community who would agree to be responsible for ongoing maintenance of the device.
• Identifying a local champion to drive community engagement.
• Encouragement to attend CPR and PAD training session provided by the supporting organisation.
• Dispelling myths such as fears of electrocution or being sued.

Respondents discussed how creating buy-in and asking communities to provide a financial contribution can provide a sense of ownership and increase the likelihood of the PAD being maintained in the long-term. One respondent stated:

*“The first step is to engage with community/business. Speak to them about the benefits of having a PAD then leave them to raise the money with the knowledge I will take care of the upkeep etc. This gives locals “ownership” and they are much more likely to attend demonstrations of CPR/PAD use and to raise funds for upkeep. Simply dropping one onto a community means they are less likely to feel engaged.”*

### Equipping the community with knowledge and skills to help someone in cardiac arrest

Respondents emphasised the importance of education and training in CPR and the use of a defibrillator so that the community feels more confident to help in an emergency. Most respondents offer free training as part of their PAD placement support package and stated that raising awareness of the locations of existing PADs in the community was an important part of this engagement. Some respondents have made willingness for communities to undergo training a prerequisite for assistance with PAD placement.

### Practical barriers and facilitators to community PAD placement

Respondents expressed the numerous factors influencing the location of PAD placement in the community. Six sub themes were identified:

### Securing consent or permission of property owner to host PAD

A key factor of placing PADs in the community is the willingness of property owners to host a PAD on their premises, participants indicated that they can often face reluctance from potential hosting sites. One respondent suggested targeting smaller, local businesses who tend to be more supportive of hosting a PAD in comparison to larger, nationwide organisations, which may face additional issues to obtain permissions.

Respondents, particularly those based in Scotland, identified challenges when working with some local authorities. These included obtaining planning permissions or access to electricity. Respondents felt that some local authorities were unwilling to support PAD placement initiatives because they did not understand the importance and benefit of PADs or had concerns about legal liabilities surrounding PAD usage and guardianship.

Respondents expressed a desire for greater support and involvement from local government bodies. Suggestions included more streamlined processes to obtain installation permission for PADs, waiving building restrictions on installing PADs, support to access power supply, encouraging businesses to allow the installation of PADs on their premises and an overall better understanding about the importance of PADs.

### Powered PAD cabinets

Respondents frequently expressed a desire to house PADs within powered cabinets in order to keep the PAD within its operating temperature range; however, finding a power source for the cabinet as well as securing consent from the property owner to provide power were identified as substantial challenges that often affected the viability of potential PAD locations.

Respondents highlighted the difficulty of identifying locations that could provide a power supply in rural or remote areas, and in particular, sourcing locations that are well-known by the community and secure to reduce the risk of theft or tampering of the PAD. Respondents acknowledged there was no easy solution to powering the cabinet and mentioned alternatives including solar-powered cabinets, cabinets that do not require a power supply, or mounting cabinets on a lamp post, which may increase the number of viable locations for PAD placement.

### Availability and accessibility

Respondents reported that ideally, PADs should be readily available day and night with the PAD ideally available 24/7 and easy to get to by potential users. Respondents primarily focused on external placement of PADs to maximise their availability and accessibility. Weather conditions that may hamper the serviceability of a PAD housed outdoors, such as extreme temperatures or rainfall are also taken into consideration for some, and PADs are placed in areas where the cabinet is less likely to be exposed to these conditions.

Respondents also highlighted the desire for PADs to be accessible from an ergonomic point of view, minimising any physical barriers for potential responders (e.g., height above the ground), and ensuring visibility of the PAD.

### PAD security

Concerns about the security of the PAD were frequently identified as affecting placement decisions. In particular, respondents expressed anxiety about theft and/or vandalism of the PAD. Respondents discussed ways to reduce community concern such as choosing locations that are less likely to experience theft or vandalism, placing the PAD in a well-lit and visible location, locations with security cameras, and placing PADs within earshot of nearby residents or staff.

The debate between housing PADs in locked versus unlocked cabinets was raised with responses reflecting the tension between keeping the PAD safe from theft or tampering but also making sure that locked cabinets did not cause critical access delays in the event of OHCA. This issue evoked strong opinions. One respondent said they would refuse to help others procure a PAD unless the cabinet is unlocked. Another respondent suggested that the introduction of a fine would help reduce likelihood of theft or vandalism and help communities overcome fears to install external PADs.

### Availability of funding

Respondents indicated that the challenge of securing funding for the procurement, installation, and ongoing maintenance costs of PADs (e.g., replacement defibrillator pads and batteries) is a barrier for potential PAD hosts. Additional installation costs associated with certain types of location - such as conservation areas or on local authority buildings - were also highlighted. Respondents reported a reliance on communities, charities, or local organisations to raise funds for equipment, to overcome this, they expressed a strong desire for public funding to support placement of PADs throughout the community and not only in locations capable of self-raising funds.

### Guardianship of PADs

Respondents discussed the importance of ensuring every PAD has an allocated guardian who is responsible for regularly checking that the device is emergency ready, replacing consumables, and getting the PAD back in an active state after use. Respondents who support the installation of PADs either assume guardianship themselves or ask the community to identify a willing individual or organisation.

## ‘Risk assessment’ methods used to estimate the need for PADs in a given location

Respondents reported using various methods of risk assessment to identify the best place to position a PAD. These included:

• Areas of high footfall and public spaces.
• Areas of high population density.
• Areas with elderly populations.
• Areas with high physical activity such as sports venues.
• Areas with delayed ambulance response.
• Locations of historical OHCAs.

Respondents also mentioned prioritising areas they felt may experience health inequalities: for example, one respondent discussed their targeted approach of placing PADs at gurdwaras for the South Asian community.

The vast majority of respondents discussed looking at existing PAD locations as a key factor in determining PAD placement. A few respondents mentioned that they had obtained information from the British Heart Foundation funded UK national defibrillator registry (The Circuit) to help determine the extent of PAD provision in their area. Respondents also considered distance from another PAD when deciding the locations of new PADs and aim to have PADs spread out in the community. Some respondents reported using target coverage metrics, such as having 95% of their residents within an estimated 300 m of a PAD, or positioning PADs to reflect a 500 m operating distance for each PAD – 500 m is the typical maximum range that ambulance services in the UK use for directing bystanders to PAD in OHCA.^23^, ^26^

Respondents indicated that PAD purchasers or benefactors may have preferences for where the PAD gets installed and this can conflict with risk assessment methods identifying areas of need.

## Discussion

This study aimed to identify factors that affect individual and organisational decision-making when placing PADs in communities, and how each factor plays a role in determining the final location of a new PAD. A common narrative across survey responses was finding the ‘right’ balance of factors, as respondents may not be able to place PADs in a location that satisfies every criterion on their list of considerations and actual placement would depend on local context.

An engaged community is essential to successfully placing PADs. Respondents believed engagement will increase the likelihood of successful fundraising for the device and the ability to secure consent to host a PAD at the chosen location, providing a power supply and long-term maintenance of the PAD. Respondents found that without meaningful engagement it is common for PADs to become unmaintained with little community awareness of the device in the event of an emergency. Successful PAD placement for many respondents does not end with the PAD installation but involves equipping the surrounding community with the knowledge, skills, and confidence to help someone in cardiac arrest.

Our findings show that people want to install PADs in locations that will maximise impact and benefit to the community. A common barrier to the acquisition of a PAD and/or cabinet in the UK is the limited availability of funding support, and the overall cost associated with PAD installation.^27^ With limited funds impacting the number of PADs that can be placed in the community, people prioritise locations where the PAD is readily available day and night, easy to access, highly visible, secure areas that decrease the likelihood of tampering or theft, external placement of PADs in powered cabinets to support accessibility and security of a PAD.

Respondents also currently use risk assessment methods to estimate the need for PADs in a given location to facilitate the best use of limited resources and benefit to communities. Some report taking a targeted approach placing PADs in deprived areas, which generally also have the highest rates of OHCA,^28^, ^29^, ^30^ have lower rates of PAD deployment,^29^, ^31^ and have poorer OHCA survival outcomes.^3^, ^32^, ^33^, ^34^ A recent study in Scotland found poor alignment between PAD and OHCA locations across levels of socioeconomic deprivation as measured by the Scottish Index of Multiple Deprivation (SIMD): in particular, OHCAs in SIMD quintile 1 (including the most deprived communities) were least likely to have PADs.^35^ Similar results have been found elsewhere in the UK and other parts of the world.^36^ Other respondents will attempt to prioritise placing PADs in locations that they estimate will benefit the greatest number of people in the community - such as areas of high footfall and population density. Respondents frequently look at current PAD provision in an area and potential gaps in provision as a proxy measure of risk and estimated need.

The International Liaison Committee on Resuscitation Scientific Statement on Public Access Defibrillation identified mathematical optimisation as an effective approach to maximising the spatiotemporal accessibility of PADs.^37^ These optimisation analyses typically involve a mathematical model that inputs historical OHCA data, existing PAD locations and pre-defined coverage range of PADs and can output modelled risk of OHCA and identify optimal locations for new PADs.^38^, ^39^ Previous optimisation models have shown ability to outperform existing PAD placement strategies and placement guidelines suggested by the American Heart Association and European Resuscitation Council.^40^, ^41^ Static optimisation models have been produced for a range of locations^35^, ^38^, ^39^, ^42^ but this information is not accessible outside of the academic literature nor applied to real-world PAD placement programmes.

The next steps in our research are to translate mathematical optimisation of PADs into a digital decision support tool for Scotland that will provide those involved in PAD placement and management freely available, on-demand information to guide effective PAD placement including risk of OHCA in communities, locations of existing registered PADs and optimal locations to install new PADs. This tool would be engineered to fit within the existing PAD placement decision-making frameworks articulated by respondents.

Additionally, we identified that PAD purchasers may have a specific location or preference for where the PAD is installed, and that this can conflict with suggestions from the supporting organisation who have identified an area of greater need. The wishes of those who raise funds for PADs should be respected and a tool providing recommendations for PAD locations using an evidence-based approach may be helpful to inform discussions about the final installation locations of PADs.

### Limitations

The majority of survey respondents were from Scotland (57%) and had low representation from Wales (7%) and Northern Ireland (4%). As such, the results may not fully reflect the PAD placement decision-making processes in other parts of the UK.

## Conclusions

Prompt bystander-initiated defibrillation can be an effective intervention to improve survival after OHCA but lack of availability of PADs is a barrier to PAD use. This study has identified key factors that influence community PAD placement decision-making processes in the UK. Decision-makers want to install PADs in locations that maximise impact and benefit to the community, but ideal locations can be constrained by various social and infrastructural factors including the relationship with the community, securing consent, limited public funding, power supply availability and support from local government bodies. Further work is required to determine how to overcome barriers to optimal community PAD placement.

## CRediT authorship contribution statement

**Diane Lac:** Conceptualization, Data curation, Formal analysis, Funding acquisition, Investigation, Methodology, Project administration, Writing – original draft. **Maria K. Wolters:** Formal analysis, Funding acquisition, Methodology, Validation, Writing – review & editing. **K.H. Benjamin Leung:** Validation, Writing – review & editing. **Lisa MacInnes:** Validation, Writing – review & editing. **Gareth R. Clegg:** Conceptualization, Funding acquisition, Methodology, Writing – review & editing.

## Declaration of Competing Interest

The authors declare that they have no known competing financial interests or personal relationships that could have appeared to influence the work reported in this paper.
