# Supplementary material for: Factors affecting public access defibrillator placement decisions in the United Kingdom: A survey study
Source: Resusc Plus. 2023 Jan 7;13:100348. doi: 10.1016/j.resplu.2022.100348 (PMC9850057; doi:10.1016/j.resplu.2022.100348)
Supplement: Supplementary data 1 [file mmc1.pdf]

PADmap: Development of a tool to guide PAD placement decisions through data-driven optimisation

---

Participant Information and Consent

Thank you for taking time to fill in this survey. Please read through the following information which will explain what kind of study we're doing, what your rights are, and what will be done with your data.

Project Title: PADmap: Improving out-of-hospital cardiac arrest (OHCA) outcomes by data-driven optimisation of placement of Public Access Defibrillators in high-risk OHCA areas.

Principal investigator: Dr Gareth Clegg

Researchers collecting data: Dr Maria Wolters and Diane Lac

Funder: Laerdal Foundation

Nature of the study.

This study aims to develop a free to use tool called PADmap to support individuals and organisations placing Public Access Defibrillators (PADs) in the community. The PADmap tool will help communities to decide where best to install a PAD by suggesting candidate locations based on historical information about where cardiac arrests are most likely to happen. The idea is that PADmap can be used by communities in conjunction with local on-the-ground knowledge to find optimal PAD locations.

Our overall aim is for PADmap to be incorporated into current PAD placement practices and to increase the availability of PADs in high risk OHCA areas. This will help enable bystanders to save more lives after OHCA.

The questionnaire will take about 15 minutes to complete.

Compensation.

You will be entered into a draw to win a £600 gift card to go towards a defibrillator cabinet of your choice if you choose to provide your email address for the draw.

Risks and Benefits.

There are no known risks to participation in this study. Your participation will help ensure that the PADmap tool is fit for purpose and can help support future PAD placement decisions for your group or organisation based on data-driven approaches.

What will happen if I decide to take part?

Once you have consented to taking part in this study, you will taken to the questionnaire that will ask you about 1) current PAD placement strategies employed by your group or organisation, 2) facilitators and barriers for PAD placement in the community, 3) the role PADmap can play in optimising PAD placement decisions, 4) experiences and attitudes towards similar types of technology and 5) demographic information from you.

What will happen to the results of this study?

The results of this study may be summarised in published articles, reports and presentations. Quotes or key findings will be anonymized: We will remove any information that could, in our assessment, allow anyone to identify you. With your consent, information can also be used for future research. Your data may be archived for a maximum of five years. All potentially identifiable data will be deleted within this timeframe if it has not already been deleted as part of anonymization.

Data protection and confidentiality.

Your data will be processed in accordance with Data Protection Law. All information collected about you will be kept strictly confidential. Your data will only be viewed by the researcher/research team (Dr Gareth Clegg, Dr Maria Wolters and Diane Lac). All electronic data will be stored on a password-protected encrypted computer, or on the University's secure encrypted cloud storage services (DataShare, OneDrive, or Sharepoint).

What are my data protection rights?

The University of Edinburgh is a Data Controller for the information you provide. You have the right to access information held about you. Your right of access can be exercised in accordance with Data Protection Law. You also have other rights including rights of correction, erasure and objection. For more details, including the right to lodge a complaint with the Information Commissioner's Office, please visit www.ico.org.uk. Questions, comments and requests about your personal data can also be sent to the

University Data Protection Officer at dpo@ed.ac.uk.

Do I have to take part?

Participation in this study is entirely up to you. You can withdraw from the study at any time, up until the anonymised data may be used for publication and dissemination purposes without giving a reason. After this point, personal data will be deleted and anonymised data will be combined such that it is impossible to remove individual information from the analysis. Your rights will not be affected. If you wish to withdraw, contact the PI. We will keep copies of your original consent, and of your withdrawal request.

Who can I contact?

If you have any further questions about the study or would like to contact the PI, please contact Diane Lac at dlac@ed.ac.uk. This study was certified according to the Informatics Research Ethics Process, RT number #6166.

If you wish to make a complaint about the study, please contact inf-ethics@inf.ed.ac.uk. When you contact us, please provide the study title and detail the nature of your complaint.

Alternative formats.

To request this document in an alternative format, such as large print or on coloured paper, please contact Diane Lac at dlac@ed.ac.uk.

Please complete the consent form below.

Please don't select more than 1 answer(s) per row.

Please select at least 6 answer(s).

	Yes	No
I have read and understood the Participant Information Sheet.	☐	☐
Questions about my participation in this study have been answered satisfactorily.	☐	☐
I am aware of the potential risks (if any).	☐	☐

I am taking part in this research study voluntarily (without coercion).	☐	☐
The anonymised data only may be shared in public research repositories.	☐	☐
I consent to take part in the above study.	☐	☐

Introduction

Public Access Defibrillators (PADs) are automated external defibrillators that are available for use by the public in the event of an out-of-hospital cardiac arrest (OHCA).

The PADmap project is developing a tool to support individuals and organisations placing PADs in the community. You have been asked to complete this questionnaire because you are involved in the process of placing PADs.

Are you...

- ○ An individual
- ○ Representing an organisation

Your Organisation

Which option best describes the organisation you represent when you are involved in placing PADs?

- Public sector - central government
- Public sector - local authority
- Public sector - NHS
- Emergency Services
- National Third sector/charitable organisation
- Local community group
- Commercial organisation
- Other

If you selected Other, please specify:

PAD Placement Locations

Where in the UK do you or your organisation place most of your PADs?

- ○ Scotland
- ○ England
- ○ Wales
- ○ Northern Ireland
- ○ We place PADs across the whole UK
- ○ We don’t place PADs

Scottish Council Areas

In which council areas do you or your organisation install PADs? Please select all the council areas that apply.

- ☐ Aberdeen City Council
- ☐ Aberdeenshire Council
- ☐ Angus Council
- ☐ Argyll and Bute Council
- ☐ City of Edinburgh Council
- ☐ Clackmannanshire Council
- ☐ Comhairle nan Eilean Siar
- ☐ Dumfries and Galloway Council
- ☐ Dundee City Council
- ☐ East Ayrshire Council
- ☐ East Dunbartonshire Council
- ☐ East Lothian Council
- ☐ East Renfrewshire Council
- ☐ Falkirk Council
- ☐ Fife Council
- ☐ Glasgow City Council
- ☐ Inverclyde Council
- ☐ Midlothian Council
- ☐ North Ayrshire Council
- ☐ North Lanarkshire Council
- ☐ Orkney Islands Council
- ☐ Perth and Kinross Council
- ☐ Renfrewshire Council
- ☐ Scottish Borders Council
- ☐ Shetland Islands Council
- ☐ South Ayrshire Council
- ☐ South Lanarkshire Council

- [ ] Stirling Council
- [ ] The Highland Council
- [ ] The Moray Council
- [ ] West Dunbartonshire Council
- [ ] West Lothian Council

PAD installation practices

A PAD ‘Guardian’ is the person or group responsible for regularly checking that the PAD is in working order, and confirming this to the ambulance service. Select all that apply to you.

- ☐ I am currently a PAD guardian
- ☐ I am supporting PAD guardians through my organisation
- ☐ I am considering purchasing and placing a PAD
- ☐ I am involved in policy making or planning PAD placement

How many years of experience do you have working with PADs?

- ○ Less than 1 year
- ○ 1-2 years
- ○ 3-5 years
- ○ 5+ years

What is the approximate number of PADs you or your organisation place annually?

- ○ Less than 5
- ○ 5-30
- ○ 31-50
- ○ 51+
- ○ We don’t place PADs

Do you or your organisation register the PADs you install with the national defibrillator network (The Circuit)?

- ○ Yes
- ○ No
- ○ We leave the decision to the PAD guardian
- ○ Other

If you selected Other, please specify:

How do you or your organisation decide where to install a PAD in the community? Select all that apply.

- ☐ We let the individual or organisation who approached us decide the installation location
- ☐ We work with the individual or organisation to make a joint decision
- ☐ We decide the location and advise those we are working with
- ☐ Other, tell us anything else which is important in your decision making process.

If you selected Other, please specify:

How often do you use the following types of information to decide the best place to install a PAD?

Please don't select more than 1 answer(s) per row.

	Never	Seldom	Sometimes	Often	Always
Footfall in an area	☐	☐	☐	☐	☐
Population density in an area	☐	☐	☐	☐	☐
Historical occurrence of cardiac arrest	☐	☐	☐	☐	☐
Local knowledge of the person/organisation purchasing the PAD	☐	☐	☐	☐	☐
Availability of a power source for a heated cabinet	☐	☐	☐	☐	☐
Safety and security of the PAD	☐	☐	☐	☐	☐
Distance from another PAD in the area	☐	☐	☐	☐	☐
Planning regulations	☐	☐	☐	☐	☐

What other types of information not specified above do you use to decide the best place to install a PAD?

Do you face any difficulties when deciding where to place PADs in the community?

- Yes
- No

PAD installation practices

You said that you face difficulties when deciding where to place PADs in the community, please tell us more.

What would help overcome these difficulties?

PAD installation practices

What are the main factors that make it easier for you to install PADs in the community?

If you have any other suggestions or comments about PAD placement that you would like to share, please type them here.

About Yourself

I identify as...

- Woman
- Man
- Non-binary / third gender
- Prefer not to say
- Prefer to self-describe

If you selected Other, please specify:

What is your age?

- 18 - 24
- 25 - 34
- 35 - 44
- 45 - 54
- 55 - 64
- 65 - 74
- >74
- Prefer not to say

What is your ethnicity?

- White: Scottish
- White: Other British

- ○ White: Irish
- ○ Mixed or Multiple Ethnic Group
- ○ Asian or Asian Scottish or Asian British
- ○ African
- ○ Caribbean or Black
- ○ Other Ethnic group, please self-describe
- ○ Prefer not to say

If you selected Other, please specify:

[   ]

[redacted]
